# Inferring person-to-person networks of *Plasmodium falciparum* transmission: are analyses of routine surveillance data up to the task?

**DOI:** 10.1186/s12936-022-04072-2

**Published:** 2022-02-21

**Authors:** John H. Huber, Michelle S. Hsiang, Nomcebo Dlamini, Maxwell Murphy, Sibonakaliso Vilakati, Nomcebo Nhlabathi, Anita Lerch, Rasmus Nielsen, Nyasatu Ntshalintshali, Bryan Greenhouse, T. Alex Perkins

**Affiliations:** 1 Department of Biological Sciences, University of Notre Dame, Notre Dame, IN USA; 2 Department of Pediatrics, University of Texas Southwestern Medical Center, Dallas, TX USA; 3 Malaria Elimination Initiative, Global Health Group, University of California, San Francisco, CA USA; 4 Department of Pediatrics, University of California, San Francisco,, CA USA; 5 National Malaria Elimination Programme, Ministry of Health, Manzini, Eswatini; 6 Department of Medicine, University of California, San Francisco, CA USA; 7 Department of Integrative Biology and Statistics, University of California, Berkeley, CA USA; 8 Clinton Health Access Initiative, Eswatini Country Office, Mbabane, Eswatini; 9 Chan Zuckerberg Biohub, San Francisco, CA USA

## Abstract

**Background:**

Inference of person-to-person transmission networks using surveillance data is increasingly used to estimate spatiotemporal patterns of pathogen transmission. Several data types can be used to inform transmission network inferences, yet the sensitivity of those inferences to different data types is not routinely evaluated.

**Methods:**

The influence of different combinations of spatial, temporal, and travel-history data on transmission network inferences for *Plasmodium falciparum* malaria were evaluated.

**Results:**

The information content of these data types may be limited for inferring person-to-person transmission networks and may lead to an overestimate of transmission. Only when outbreaks were temporally focal or travel histories were accurate was the algorithm able to accurately estimate the reproduction number under control, *R*_*c*_. Applying this approach to data from Eswatini indicated that inferences of *R*_*c*_ and spatiotemporal patterns therein depend upon the choice of data types and assumptions about travel-history data.

**Conclusions:**

These results suggest that transmission network inferences made with routine malaria surveillance data should be interpreted with caution.

**Supplementary Information:**

The online version contains supplementary material available at 10.1186/s12936-022-04072-2.

## Background

Concomitant with improved epidemiological surveillance, there is growing interest to leverage the collected data to infer transmission networks for a wide range of pathogens and to use those inferences to inform public health efforts. Past studies have incorporated temporal data [1] and spatial data [2–5] to estimate pairwise probabilities of transmission between individual cases and to use those estimates to infer time-varying and spatially varying reproduction numbers, respectively. More recently, methods have been developed to incorporate this type of detailed, individual-level epidemiological data [6–8] to infer transmission networks for infectious diseases of humans, including severe acute respiratory syndrome [9] and tuberculosis [10], and of animals, such as rabies [11] and foot-and-mouth disease [12].

In addition to the diseases for which these methods have been applied to date, there is a growing need to apply similar methods to malaria in near-elimination settings. As incidence of malaria declines within a country, transmission becomes more heterogeneous in space and time [13]. Focal areas of high transmission, known as ‘hotspots’, pose a serious risk of fuelling resurgence if left untargeted, potentially reversing decades of progress towards elimination [14]. To this end, granular estimates of when and where transmission occurs are needed, as spatially aggregated estimates may obscure important heterogeneities of practical relevance to control efforts [15]. In addition to characterizing details of local transmission, measurement of progress towards malaria elimination hinges on correct classification of cases as imported (i.e., acquired outside the country) or locally acquired [16, 17], which is a byproduct of estimating transmission networks.

Previous work on malaria has made progress on the use of individual-level epidemiological data to infer transmission networks and reproduction numbers of *Plasmodium falciparum*, the parasite primarily responsible for human malaria in many regions of the world. Churcher et al. [18] used temporal data to estimate the proportion of imported cases needed to confidently estimate the reproduction number under control, *R*_*c*_, below one and thereby provide evidence of controlled, non-endemic malaria transmission. Reiner et al. [6] then built upon this work by incorporating spatial data and inferring an individual-level transmission network of *P. falciparum* in Eswatini. More recently, Routledge et al. [19, 20] used related approaches to infer transmission networks and *R*_*c*_ of *Plasmodium vivax* in El Salvador and China.

As the adoption of these methods increases, in particular for malaria, care should be taken to assess how the epidemiological setting and the inclusion or exclusion of certain data types might affect the accuracy of transmission network inferences, as well as resultant inferences about epidemiological quantities, including *R*_*c*_ and spatiotemporal variation therein. A recent study by Campbell et al. [21] noted that epidemiological data alone were generally insufficient to reconstruct transmission networks of other pathogens, ranging from *Mycobacterium tuberculosis* to SARS-CoV. Although falciparum malaria was not considered in that analysis, its long serial interval [22] calls into the question the utility of epidemiological data for this purpose, though this has been largely unaddressed in past studies. Furthermore, past transmission network inferences for malaria have relied on various types of epidemiological data, ranging from the timing of symptom onset [19, 20] to more detailed spatiotemporal data [6]. Each study incorporated travel-history information into transmission network inferences and considered these data to be perfectly accurate, assuming that all cases that reported travel were imported. However, travel history may be an imperfect indicator of importation owing to errors in recall [17] and the fact that travel to an area of ongoing transmission alone does not guarantee that an individual was infected there [17, 23]. *Plasmodium falciparum* transmission network inferences are likely to be sensitive to the choice of data types [24], and failure to evaluate the sensitivity of transmission network inferences to choices about data types and different assumptions about possible errors in travel-history data could lead to apparently confident, though ultimately incorrect, assessments of *P. falciparum* transmission risk in near-elimination settings.

Here, a Bayesian method for inferring transmission networks based on temporal, spatial, and travel-history data for individual malaria cases is used to characterize the sensitivity of transmission network inferences to the inclusion of different data types and to different assumptions about the accuracy of travel histories. This method builds upon previous work by leveraging individual-level epidemiological data to obtain posterior estimates of transmission networks and model parameters in a way that can accommodate different assumptions about errors in travel histories. After establishing a proof-of-concept of the inference method on simple test cases, the method was applied to real-world surveillance data from Eswatini and additional simulated data sets to understand how the inclusion or exclusion of different data types and different assumptions about travel-history error affect the ability to infer transmission networks and estimate transmission metrics, namely *R*_*c*_.

## Methods

### Bayesian framework for estimating transmission linkages

**Fig. 1:**
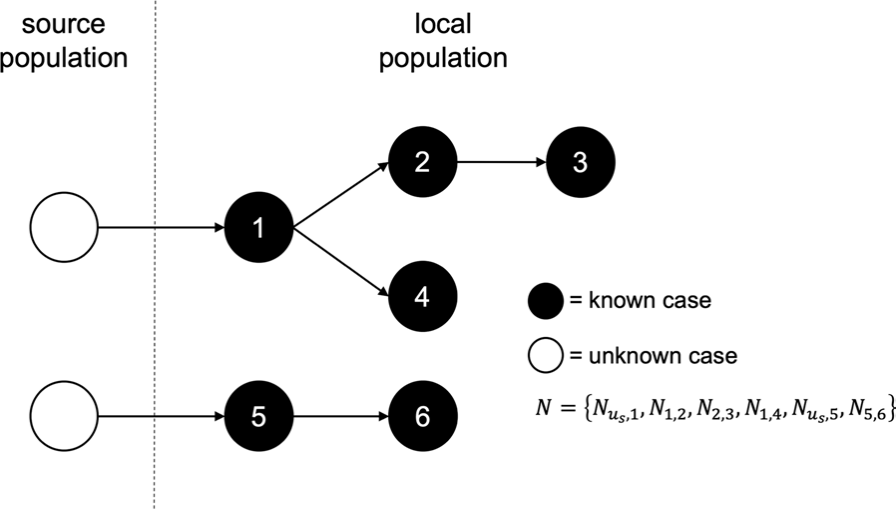
Schematic of a hypothetical transmission network A hypothetical transmission network is presented along with the corresponding notation. In the schematic, white circles denote unobserved cases, and black circle denote observed cases. Arrows represent transmission between two cases

The goal was to obtain probabilistic estimates of a transmission network *N* that defines transmission linkages among a set of known cases. The transmission network is defined as a directed, acyclic graph comprised of a set of directed edges represented as *N* = {*N*_*i,j*_} for all *i*, *j*. Each *N*_*i,j*_ indicates that case *i* is hypothesized to contain parasites that are the most direct observed ancestors of the parasites contained in case *j*. In addition, at least one edge denoted *N*_*u,j*_ must exist in the network, indicating that the parasites contained in case *j* have no ancestors among the parasites contained in any known local case and are instead contained in some unknown case *u* from some source population *s*, such that it is denoted *u*_*s*_. To illustrate this terminology, an example transmission network is depicted in Fig. 1.

To estimate *N*, the algorithm used spatial, temporal, and travel-history data about all cases, denoted as $\vec{X}_{s}$, ${X}_{t}$, and ${X}_{h}$, respectively. It did so within a Bayesian statistical framework, meaning that it sought to estimate the joint posterior probability density,

1 $$\mathrm{Pr}\left(N,\Theta |{X}_{t},\vec{X}_{s},{X}_{h}\right)=\frac{\mathrm{Pr}\left({X}_{t},\vec{X}_{s},{X}_{h}|N,\Theta \right)\mathrm{Pr}(\mathrm{N},\Theta )}{\mathrm{Pr}({X}_{t},\vec{X}_{s},{X}_{h})},$$

of the transmission network defined by *N* and the model parameters Θ conditional on the data $\vec{X}_{s}$, ${X}_{t}$, and ${X}_{h}$. The first term in the numerator of Eq. (1) is the likelihood of *N* and Θ conditional on the data. The second term in the numerator is the prior probability of *N* and Θ. The term in the denominator is the probability of the data, which is an intractable quantity to calculate directly given that it would require evaluation of an extremely high-dimensional integral over *N* and Θ. To address this, a Markov chain Monte Carlo algorithm was used to draw random samples of *N* and Θ from the posterior distribution specified in Eq. (1).

The most critical piece of the inference framework is the likelihood, which was defined as a function of each case *j* as

2 $$\mathcal{L}(N,\Theta {|X}_{t},\vec{X}_{s},{X}_{h})=\prod_{j}\mathrm{Pr}\left({X}_{t,j},\vec{X}_{s,j},{X}_{h,j}|{N}_{.,j},\Theta \right).$$

Below, the probability of the data associated with each known case *j* as a function of different assumptions that are possible about how case *j* is connected to the rest of the transmission network is defined.

### Scenario 1: Local transmission between known cases *i* and *j*

When case *i* contains parasites that are immediate ancestors of the parasites contained in case *j*, its contribution to the likelihood is represented as

3 $$\mathrm{Pr}\left({X}_{t,j},\vec{X}_{s,j},{X}_{h,j}|{N}_{i,j},\Theta \right)=\mathrm{Pr}\left({X}_{t,j}|{N}_{i,j},\Theta \right)\mathrm{Pr}\left(\vec{X}_{s,j}|{{X}_{t,j}, N}_{i,j},\Theta \right)\mathrm{Pr}\left({X}_{h,j}|{N}_{i,j},\Theta \right),$$

which is the product of the probabilities of the temporal, spatial and travel-history data given the network and model parameters. This formulation assumes that those data are generated independently for each individual, with the exception of a dependence of the spatial data on the temporal data.

### Probability of the temporal data

To characterize the time elapsed between two cases resulting from local transmission, a model of the generation and serial intervals for *P. falciparum* malaria by Huber et al. [22] was used. The generation interval represents the time between infection of a primary and secondary case, whereas the serial interval represents the time between detection of those cases. Because the timing of infection per se (i.e., an infectious mosquito inoculating a susceptible human) is typically unknown, this study focused on the serial interval as the most apropos temporal quantity relating cases.

In deriving the probability of a given length of the serial interval, Huber et al. [22] convolved a discrete random variable representing variability in the generation interval (*GI*) with a discrete random variable representing variability in the time between infection with *P. falciparum* and detection by surveillance, i.e., the infection to detection period (*IDP*). That framework yielded

4 $$\mathrm{Pr}\left({SI}_{i,j}=-a+b+c\right)=\sum_{a}\sum_{b}\sum_{c}\mathrm{Pr}\left({IDP}_{i}=a\right)\mathrm{Pr}\left({GI}_{i,j}=b\right)\mathrm{Pr}\left({IDP}_{j}=c\right){\mathbb{I}}\left(x=-a+b+c\right),$$

as the probability of a serial interval of length *SI*_*i*,*j*_. The algorithm allowed for different models of the serial interval depending upon differences in the *GI* and *IDP* for different types of primary and secondary cases. For instance, the mean *GI* for a primary infection receiving treatment was 48.4 days, compared to 101.6 days for an untreated primary infection. Furthermore, symptomatic cases were assumed to present in a clinic some number of days after infection as informed by empirical data from Zanzibar with a mean of 16.6 days [22]. For an asymptomatic infection, it was assumed that detection occurred through active surveillance at a randomly drawn day among all days where its asexual parasitaemia exceeds a detection threshold, resulting in a mean of 69.8 days [22]. The choice of *IDP* for both the primary and secondary case informs the probability of two cases separated in time by ${SI}_{i,j}={X}_{t,j}-{X}_{t,i}$ days.

### Probability of the spatial data

Following Reiner et al. [6], it was assumed that a simple two-dimensional Wiener diffusion process determines the location of secondary cases relative to the location of their associated primary case. It follows that, for a given diffusion coefficient *D* with units *km*^*2*^*day*^*−1*^ and generation interval *GI*_*i,j*_, the two-dimensional location $\vec{X}_{s,j}$ of the secondary case *j* is described by a bivariate normal distribution with probability density

5 $$f\left(\vec{X}_{s,j} |\vec{X}_{s,i},D,{GI}_{i,j},{N}_{i,j},\Theta \right)=\frac{1}{2\pi {\sigma }^{2}\left({GI}_{i,j}\right)}{e}^{-\frac{\Vert \vec{X}_{s,j}-\vec{X}_{s,i}\Vert }{2{\sigma }^{2}\left({GI}_{i,j}\right)}},$$

where ${\sigma }^{2}\left({GI}_{i,j}\right)=D{GI}_{i,j}$. This formulation assumes that each spatial dimension is independent, that the variance scales linearly with the generation interval, and that movement is isotropic across a continuous landscape. By making the spatial scale of transmission dependent upon time, the algorithm assumed that a longer generation interval provides a longer period of time over which movement of the primary case could occur. This permitted transmission linkages farther apart in space as the length of the generation interval increased. Because mosquito movement is more restricted and could lead to shorter transmission distances than would be obtained using the two-dimensional Wiener diffusion process, this study evaluated the sensitivity of the inferences to this assumption in the Supplement by using a time-invariant exponential kernel [25].

One complication to Eq. (5) is that the generation interval *GI*_*i,j*_ is unobserved and, therefore, cannot take on a fixed value. Instead, data about the serial interval *SI*_*i,j*_ must be used to inform the generative model for $\vec{X}_{s,j}$. To do so, the algorithm takes advantage of the property of normal random variables that the sum of two or more random variables is itself a normal random variable [26]. This property allows for the recasting of Eq. (5) as a function of *SI* rather than *GI* by computing the appropriate σ^2^ as

6 $${\sigma }^{2}\left(SI\right)=\int {\sigma }^{2}\left(GI\right)\mathrm{Pr}\left(GI|SI\right)dGI,$$

which is effectively a weighted sum of the spatial variances associated with a given *GI* proportional to the probability that the generation interval is exactly *GI* days given that the serial interval was observed to be *SI* days. This results in

7 $$f\left(\vec{X}_{s,j} |\vec{X}_{s,i},D,{SI}_{i,j},{N}_{i,j},\Theta \right)=\frac{1}{2\pi {\sigma }^{2}\left({SI}_{i,j}\right)}{e}^{-\frac{\Vert \vec{X}_{s,j}-\vec{X}_{s,i}\Vert }{2{\sigma }^{2}\left({SI}_{i,j}\right)}},$$

as the probability density of the spatial data that was assumed.

In the event that case *i* has missing spatial data, one cannot compute the spatial likelihood of Eq. (7). To address this, a latent unobserved quantity ${\tilde{X }}_{s,i}$ was defined, which represents the unknown location of case *i*. The algorithm then integrated over the uncertainty in ${\tilde{X }}_{s,i}$,

8 $$f\left(\vec{X}_{s,j}|D, S{I}_{i,j},{N}_{i,j},\Theta \right)=\int f\left(\vec{X}_{s,j}|{\tilde{X }}_{s,i},D,{N}_{i,j},\Theta \right)f\left({\tilde{X }}_{s,i}|\vec{X}_{s,j},D\right)d{\tilde{X }}_{s,i},$$

to compute the probability density of case *j* with known spatial location $\vec{X}_{s,j}$ arising from case *i* with unknown spatial location ${\tilde{X }}_{s,i}$. Equation (8) is computed as the product of the probability density of the location of a known case *j* conditional on an unknown location ${\tilde{X }}_{s,i}$ and the probability density of spatial separation ${\vec{X}_{s,j}-\tilde{X }}_{s,i}$ conditional on the diffusion coefficient *D* for all ${\tilde{X }}_{s,i}$. Because it was assumed that movement is isotropic, Eq. (8) is a two-dimensional Gaussian integral, simplifying to

9 $$f\left(\vec{X}_{s,j}|D, S{I}_{i,j},{N}_{i,j},\Theta \right)=\frac{1}{4\pi {\sigma }^{2}(S{I}_{i,j})}.$$

In the event that case *j* has missing spatial data and case *i* has known spatial data, the latent unobserved quantity becomes ${\tilde{X }}_{s,j}$. The algorithm then integrates over the uncertainty in ${\tilde{X }}_{s,j}$ and calculates $f\left(\vec{X}_{s,j}|D, S{I}_{i,j},{N}_{i,j},\Theta \right)$ using Eq. (8–9).

### Probability of the travel-history data

Although it was assumed in this scenario that a person’s infection was locally acquired, the model must still be capable of explaining the travel-history data *X*_*h,j*_. Thus, τ_l_ is the probability that case *j* reported travel (i.e., *X*_*h,j*_ = 1) even though they were not infected during that period of travel, such that

10 $${\text{Pr}}\left( {X_{{h,j}} |N_{{i,j}} ,\Theta } \right) = \left\{ {\begin{array}{*{20}c} {\tau _{l} ,\quad X_{{h,j}} = 1} \\ {1 - \tau _{l} ,\quad X_{{h,j}} = 0} \\ \end{array} } \right..$$

In the event that case *j* has missing travel-history data, the travel-history likelihood of Eq. (10) cannot be computed. To address this, a latent unobserved quantity ${\tilde{X }}_{h,j}$, which represents the unknown travel history of case *j*, was defined. The algorithm then sums across the uncertainty in ${\tilde{X }}_{h,j}$,

11 $$\mathrm{Pr}\left({X}_{h,j}=\mathrm{NA}|{N}_{i,j},\Theta \right)=\mathrm{Pr}\left({\tilde{X }}_{h,j}=1\right){\tau }_{l}+\left(1-\mathrm{Pr}\left({\tilde{X }}_{h,j}=1\right)\right)\left(1-{\tau }_{l}\right),$$

to compute the probability that case *j* was locally acquired given an unknown travel history. In Eq. (11), $\mathrm{Pr}\left({\tilde{X }}_{h,j}=1\right)$ was computed as the proportion of cases with a positive travel history among all cases with known travel-history data.

Taken together with the probabilities of the temporal and spatial data described above, the product of these three probabilities constitutes the entirety of the contribution of a case *j* infected by a known local case *i* to the overall likelihood of *N* and Θ.

### Scenario 2: Importation of local case *j* from source population *s*

In the event of ${N}_{{u}_{s},j}$, the contribution of such a case to the overall likelihood of *N* and Θ is represented as the product of the probabilities of its temporal, spatial and travel-history data under similar assumptions as in Scenario 1. The key difference in this scenario is that there is no information about the unknown source case that gave rise to case *j*.

### Probability of the temporal data

Because the person containing parasites that are the direct ancestors of those in case *j* is unobserved and does not have an *X*_*t,i*_, the probability of the temporal data as described in Scenario 1 cannot be computed. It is important though to obtain a probability comparable to that from Scenario 1 as a reference point for determining whether it is more likely that a given case arose from some other known local case or from an unknown case *u*_s_ from source population *s*. To do so, the algorithm considers the variable ${\tilde{X }}_{t,{u}_{s}}$, which is a latent variable describing the timing of when *u*_s_ would have been detected, had it been detected.

Because *u*_s_ is not observed, it was considered to be asymptomatic and untreated. The algorithm then calculated the probability of the timing of a known case *j* arising from an unknown case *u*_s_ as

12 $$\mathrm{Pr}\left({X}_{t,j}|{N}_{{u}_{s},j},\Theta \right)=\int \mathrm{Pr}({X}_{t,j}\left|{\tilde{X }}_{t,{u}_{s}},{N}_{{u}_{s},j},\Theta \right)\mathrm{Pr}\left(\mathrm{SI}={X}_{t,j}-{\tilde{X }}_{t,{u}_{s}}\right)d{\tilde{X }}_{t,{u}_{s}},$$

by integrating over uncertainty in ${\tilde{X }}_{t,{u}_{s}}$. This is represented as the product of the probability of the timing of a known case *j* conditional on an unknown time of detection ${\tilde{X }}_{t,{u}_{s}}$ and the probability of the serial interval ${X}_{t,j}-{\tilde{X }}_{t,{u}_{s}}$ for all ${\tilde{X }}_{t,{u}_{s}}$. Equation (12) does not distinguish between symptomatic and asymptomatic cases *j* because the calculation is identical; only the serial interval distributions differ.

### Probability of the spatial data

Without an ${\tilde{X }}_{t,{u}_{s}}$ for the unobserved case *u*_s_, the algorithm lacked information on the serial interval between it and case *j*. Consequently, the probability from Eq. (7) could not be used in that particular form. Instead, the spatial variance was computed as a function of the diffusion coefficient alone, yielding

13 $${\sigma }^{2}\left(D\right)=\int D GI \mathrm{Pr}\left(GI\right) dGI.$$

Equation (13) integrates across all possible generation intervals and simplifies to $D{\mathbb{E}}\left[GI\right]$, the product of the diffusion coefficient and the expectation of the generation interval.

This spatial variance was applied to the unobserved latent variable ${\tilde{X }}_{s,{u}_{s}}$, which represents the unknown location of the unobserved case *u*_s_. The algorithm integrated over uncertainty in ${\tilde{X }}_{s,{u}_{s}}$ to compute the probability density,

14 $$f\left({X}_{s,j}|D,{N}_{{u}_{s},j},\Theta \right)=\int f\left({X}_{s,j}|{\tilde{X }}_{s,{u}_{s}},D,{N}_{{u}_{s},j},\Theta \right)f\left({\tilde{X }}_{s,{u}_{s}}|{X}_{s,j},D\right)d{\tilde{X }}_{s,{u}_{s}},$$

of the location of a known case *j* arising from an unknown source case *u*_s_ with unknown location ${\tilde{X }}_{s,{u}_{s}}$. This is represented as the product of the probability density of the location of a known case *j* conditional on an unknown location ${\tilde{X }}_{s,{u}_{s}}$ and the probability density of spatial separation ${{X}_{s,j}-\tilde{X }}_{s,{u}_{s}}$ conditional on the diffusion coefficient *D* for all ${\tilde{X }}_{s,{u}_{s}}$. As in Eq. (9), Eq. (14) was treated as an evaluation of the Gaussian integral, evaluating to

15 $$f\left({X}_{s,j}|D,{N}_{{u}_{s},j},\Theta \right)=\frac{1}{4\pi D{\mathbb{E}}\left[GI\right]}.$$

In Eq. (15), *D* is the diffusion coefficient and ${\mathbb{E}}\left[GI\right]$ is the expectation of the generation interval.

### Probability of the travel-history data

The travel history *X*_*h,j*_ was considered to be a binary variable with a value of 1 indicating a presumed malaria importation due to reported international travel to an area with known malaria transmission within the past eight weeks but excluding the minimum incubation period of one week prior to the data of presentation. After defining the probability τ_s_ that ${X}_{h,j}=1$ conditional on ${N}_{{u}_{s},j}$, it follows that

16 $$\mathrm{Pr}\left({X}_{h,j}|{N}_{{u}_{s},j},\Theta \right)=\left\{\begin{array}{c}{\tau }_{s}, { X}_{h,j}=1\\ {1-\tau }_{s}, {X}_{h,j}=0\end{array}\right.,$$

which constitutes the contribution of the travel history of such a case to the overall likelihood of *N* and Θ. If the travel history of case *j* is unknown, an analogous calculation to Eq. (11) is made using τ_s_.

## Bayesian inference

### Markov Chain Monte Carlo algorithm

To avoid evaluating the high-dimensional integral over *N* and Θ, samples of *N* and Θ were drawn from their posterior distribution defined by Eq. (1) using a Metropolis–Hastings Markov chain Monte Carlo (MCMC) method [27, 28]. To begin the chain, *N* and Θ were initialized to *N*^(1)^ and Θ^(1)^, and each subsequent step *i* in the chain was denoted *N*^(*i*)^ and Θ^(*i*)^. At each step, states *N*^′^ and Θ^′^ were proposed with $\mathrm{Pr}\left(\left({N}^{\left(i\right)},{\Theta }^{\left(i\right)}\right)\to \left({N}^{^{\prime}},{\Theta }^{^{\prime}}\right)\right)$. Proposed states were accepted with probability

17 $${\alpha }_{\mathrm{update}}=\mathrm{min}\left[1,\frac{\pi \left({N}^{^{\prime}},{\Theta }^{^{\prime}}\right)\mathrm{Pr}\left(\left({N}^{^{\prime}}, {\Theta }^{^{\prime}}\right)\to \left({N}^{\left(i\right)},{\Theta }^{\left(i\right)}\right)\right)}{\pi \left({N}^{\left(i\right)},{\Theta }^{\left(i\right)}\right)\mathrm{Pr}\left(\left({N}^{\left(i\right)}, {\Theta }^{\left(i\right)}\right)\to \left({N}^{^{\prime}}, {\Theta }^{^{\prime}}\right)\right)}\right],$$

where $\pi (N,\Theta )$ is the product of the likelihood $\mathrm{Pr}(\vec{X}_{s},{X}_{t},{X}_{h}|N,\Theta )$ of *N* and Θ conditional on the data and the assumed prior probability $\mathrm{Pr}(N,\Theta )$ of *N* and Θ. After a random draw *R* from a uniform distribution, the chain was updated according to

18 $${N}^{(i+1)},{\Theta }^{(i+1)}=\left\{\begin{array}{c}{N}^{^{\prime}},{\Theta }^{^{\prime}}, \quad R\le \alpha \\ {N}^{\left(i\right)},{\Theta }^{(i)}, R>\alpha \end{array}\right..$$

To reduce the probability of the chain becoming stuck at a local maximum, this study employed Metropolis-coupled Markov chain Monte Carlo (MC^3^) [29]. Implementing MC^3^ involved running multiple chains in parallel, with ${\pi }_{c}(N,\Theta )$ in chain *c* raised to the power *β*_c_ according to

19 $${\beta }_{c}=1+\lambda \left(c-1\right),$$

where λ > 0 is a temperature increment parameter that governs the degree to which each chain is ‘heated’. As a result of setting *β*_1_ = 1 $,$ ${\pi }_{1}(N,\Theta )$ is directly proportional to the joint posterior distribution and is referred to as the master or ‘cold’ chain. This algorithm effectively flattens the likelihood in the heated chains by setting *β*_c_ > 1, allowing them to explore the parameter space more freely and to encounter alternative high-density regions more readily than the cold chain would alone. At a pre-defined frequency, two randomly selected chains *i* and *j* were allowed to swap parameter sets according to a swap probability

20 $${\alpha }_{\mathrm{swap}}=\mathrm{min}\left[1, \frac{\pi {\left({N}^{\left(j\right)},{\Theta }^{\left(j\right)}\right)}^{{\beta }_{i}}\pi {\left({N}^{\left(i\right)},{\Theta }^{\left(i\right)}\right)}^{{\beta }_{j}}}{\pi {\left({N}^{\left(i\right)},{\Theta }^{\left(i\right)}\right)}^{{\beta }_{i}}\pi {\left({N}^{\left(j\right)},{\Theta }^{\left(j\right)}\right)}^{{\beta }_{j}}}\right],$$

where $\pi (N,\Theta )$ is the same as it was in Eq. (17). A swap into the master chain only occurred if it was from one of the two randomly selected chains and $R\le {\alpha }_{\mathrm{swap}}$. This analysis recorded a total of 100 million samples from the posterior distribution, discarding the first 50 million samples as burn-in and thinning the chain every 10,000 samples between each recorded sample.

### Proposals

Proposals made by the MC^3^ algorithm involved changes to the parameters (i.e., *D*, τ_s_, and τ_l_) and changes to the transmission network topology. Each proposal occurred with a fixed probability, where the sum of these proposal probabilities was equal to one.

Proposals to change parameters involved updating *D*, τ_s_, or τ_l_. To update the value of *D*, a new value was drawn from a normal distribution with mean set to the current value of the parameter and variance set to 2.5. Values of *D* proposed must be strictly non-negative, so any proposed *D* that was less than zero was rejected and assigned ${\alpha }_{\mathrm{update}}=0$. Similarly, new values of τ_s_ and τ_l_ were chosen according to normal distributions with means set to their current parameter value and variance set to 0.25. Because τ_s_ and τ_l_ are probabilities, any proposed value that fell outside the range [0,1] was rejected and assigned ${\alpha }_{\mathrm{update}}=0$.

Changes proposed to the network topology involved the addition or removal of an ancestor from a randomly selected node. The algorithm assigned a uniform probability of proposing case *a* as an ancestor to a randomly selected case *i*, such that proposals to the network topology are uninformed by spatial and temporal data. Each proposed ancestor was chosen from the set of ancestors that would ensure that the network remained acyclic. Furthermore, the proposal probability of removing case *a* as an ancestor to a randomly selected case *i* was defined as

21 $$\mathrm{Pr}\left(\mathrm{remove} a\right)={\overline{A} }_{i}^{-1},$$

where ${\overline{A} }_{i}$ represents the size of set *A*_i_ of all ancestors to case *i*. Proposed changes to the network are then accepted according to Eq. (17).

### Prior assumptions

Strong priors were placed on τ_s_ and τ_l_, because it was assumed that travel histories were mostly, but not completely, accurate. The algorithm used a beta-distributed prior on τ_s_, with parameters ${\alpha }_{{\tau }_{s}}=12$ and ${\beta }_{{\tau }_{s}}=3$, which resulted in a mean of 0.8 and a variance of 0.01 for this prior distribution. The algorithm also used a beta distributed prior on τ_l_, with parameters ${\alpha }_{{\tau }_{l}}=3$ and ${\beta }_{{\tau }_{l}}=12$, which resulted in a mean of 0.2 and a variance of 0.01. A uniform prior on *D* over the interval $\left[{10}^{-3},\infty \right)$ and an even prior across all possible network configurations were assumed, meaning that those prior probabilities cancelled out in eqs. (17) and (20).

### Assessing convergence

For *D*, τ_s_, and τ_l_, convergence was assessed using the Gelman-Rubin statistic [30], with values below 1.1 indicating convergence. For the transmission network *N*, convergence was assessed by calculating correlation coefficients of case-level probabilities across five chains from independent realizations of the MC^3^ algorithm, for a total of 10 pair-wise comparisons across the five chains. The two case-level probabilities considered were the posterior probability that each case was infected by an unknown case *u*_*s*_ from a source population and the posterior probability that each case *j* was infected by each other case *i*. Higher values of these correlation coefficients provided stronger support for convergence.

## Results

To establish proof-of-concept, this study first applied the inference method on three simple test cases and evaluated how well the inferences recovered the true transmission networks. Then, the method was applied to surveillance data collected in Eswatini during 2013–2017. The focus was less on understanding malaria epidemiology in Eswatini and more on understanding how epidemiological conclusions change with the inclusion or exclusion of different data types and different assumptions about travel histories. These inference settings used: (1) spatial and temporal data while estimating the accuracy of the travel history (default setting); (2) spatial and temporal data while believing the travel history; (3) spatial and temporal data alone; (4) temporal data while estimating the accuracy of the travel history; and, (5) temporal data while believing the travel history. To validate the inferences based on data from Eswatini, simulated data was generated using posterior parameter estimates obtained from the data from Eswatini and evaluated the ability of our inference method to recover the true transmission networks along with the underlying parameters on those simulated data. Finally, a simulation sweep across different epidemiological settings was performed to determine the range of conditions under which our inference method yielded reliable estimates of transmission. A full description of the analyses and additional results can be found in the Supplement.

### Application to Eswatini surveillance data

**Fig. 2:**
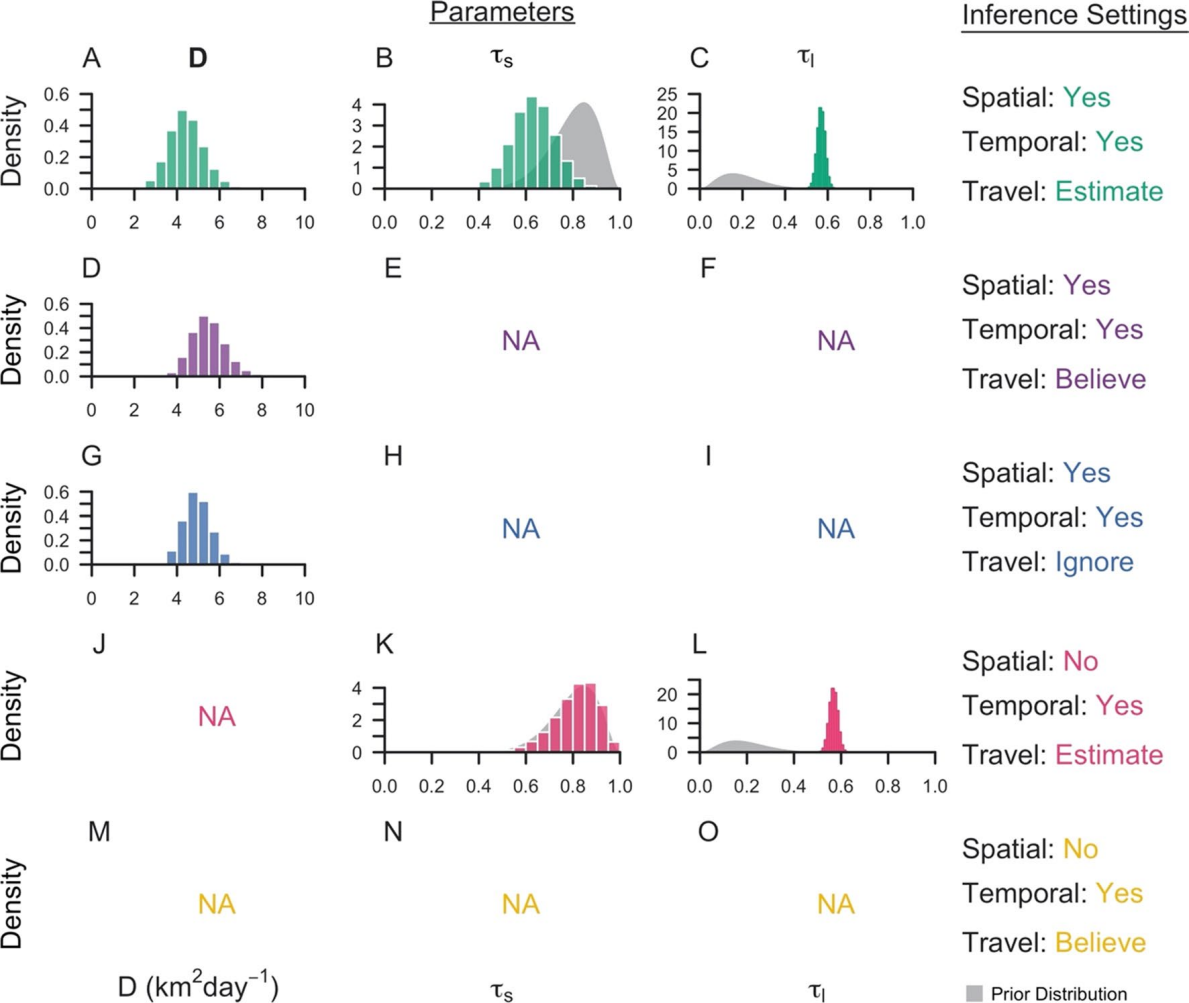
Marginal posterior distributions of parameters from Eswatini surveillance data. Histograms represent the marginal posterior distribution of each parameter, colour-coded by the inference settings used. D is the diffusion coefficient with units sq km day^−1^, τ_s_ is the probability that an imported case reports travel, and τ_l_ is the probability that a locally acquired case reports travel. Grey shapes represent the prior distributions placed on each parameter. Inference settings in which a given parameter was not estimated are indicated by NA

**Fig. 3:**
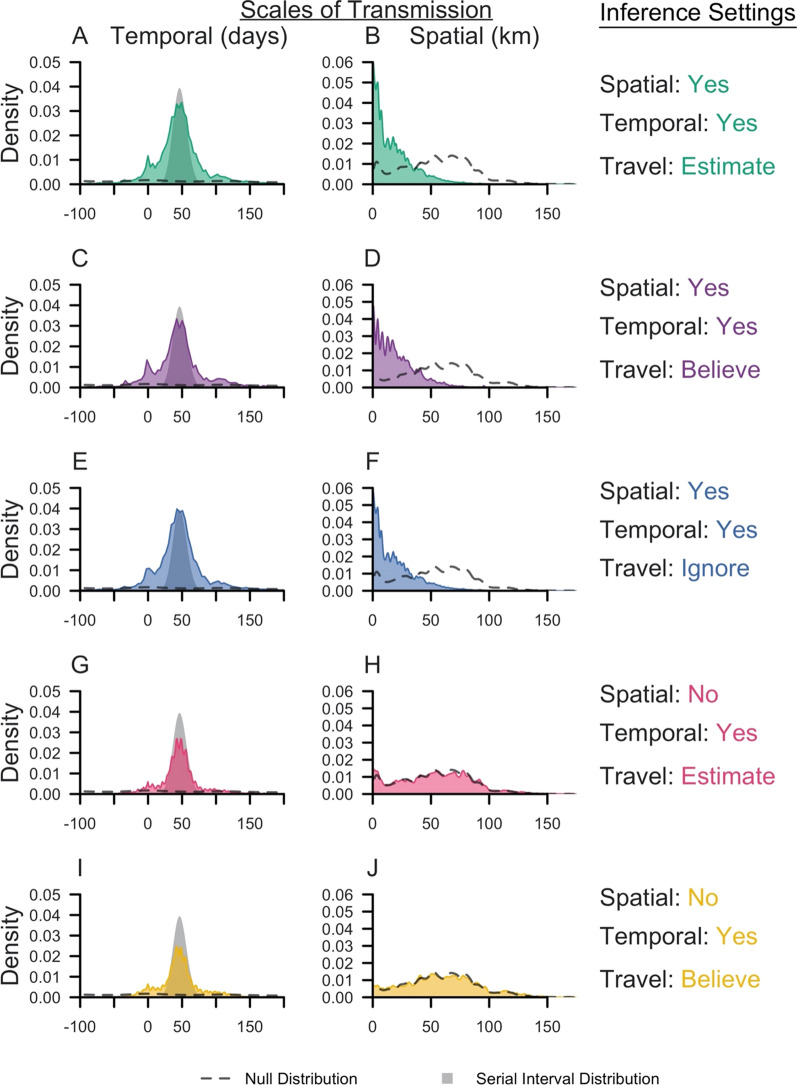
Spatial and temporal scales of transmission in Eswatini. Kernel density plots of the spatial (km) and temporal (days) scales of transmission are reported and colour-coded for each inference setting. Dashed lines indicate the corresponding null distribution, generated from all random pairs of cases in the Eswatini surveillance data set. The null distribution was different if we believed the travel history, because classification of cases on the basis of travel history reduced the pairs of cases that could be randomly sampled. The grey shape is the serial interval distribution used in the likelihood

**Fig. 4:**
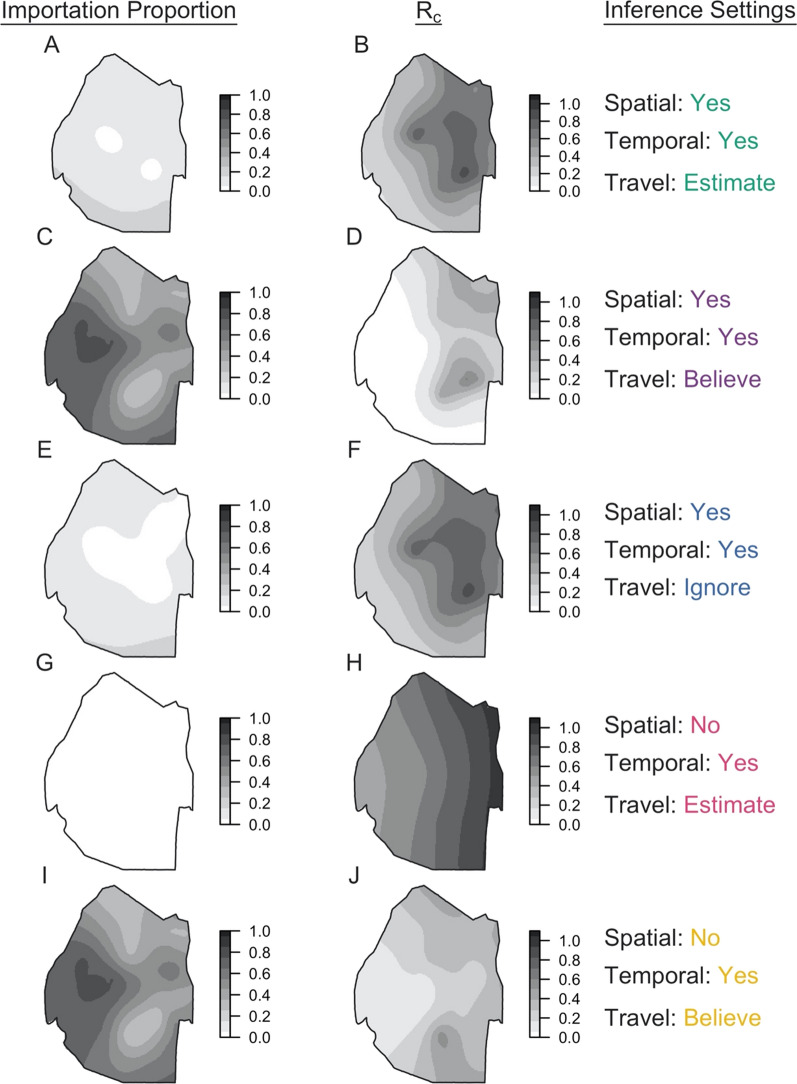
Spatial distribution of importation and transmission risk in Eswatini. Maps of the proportion of cases that are imported and the reproduction number under control (R_c_) were generated for each inference setting using a generalized additive model with a Gaussian process basis function setting using the mgcv package in R [50, 51]. In each plot, darker colours indicate greater importation or transmission risk

The method was applied to surveillance data collected in Eswatini during 2013–2017. Under the default inference setting, the median posterior diffusion coefficient *D*, which quantifies the spatial spread of transmission, was estimated to be 4.40 sq km day^−1^ (95% Credible Interval: 2.93–6.13 sq km day^−1^) (Fig. 2A). This corresponded to a median inferred transmission distance of 13.0 km (0.0130–64.8 km), a median inferred serial interval of 45 days (−37–148 days) (Fig. 3A, B), and median estimates of τ_s_, the probability that an imported case reported travel, of 0.63 (0.46–0.81) compared to the prior distribution mean of 0.80 and τ_l_, the probability that a locally acquired case reported travel, of 0.57 (0.53–0.61) compared to the prior distribution mean of 0.20 (Fig. 2B, C). That the 95% credible interval for τ_s_ contained 0.50 indicated that the inference algorithm found limited use of travel-history data in discriminating between imported and locally acquired cases, because that implies that imported cases have equal probabilities of reporting or not reporting travel. The algorithm estimated the proportion of imported cases to be 0.046, corresponding to *R*_*c*_ = 0.95. Mapping risk of importation and local transmission across Eswatini under the default inference setting, the algorithm estimated consistently low risk of importation throughout the country and transmission hotspots in the northeastern part of Eswatini, close to the border with Mozambique (Fig. 4A, B).

Parameter estimates and transmission network inferences differed under other inference settings. When the travel history was believed, a larger median transmission distance was estimated (Fig. 3D). This increase in the spatial scale of transmission can be attributed to clusters of cases with positive travel histories located near metropolitan areas. By forcing those cases to be imported, the algorithm tended to infer transmission across longer distances to explain the origins of the remainder of cases that did not report travel and were thereby inferred to be locally acquired. With respect to time, all five inference settings produced consistent serial interval estimates, although the inclusion of spatial data allowed for a wider range of transmission linkages in time (Fig. 3A, C, E). Finally, in the absence of spatial data, the model estimated higher predictive power of travel histories in identifying imported cases (τ_s_: 0.83, [0.60, 0.95]), though the travel history was consistently found to be uninformative for identifying locally acquired cases (τ_l_: 0.57, [0.53, 0.60]) (Fig. 2K, L).

**Fig. 5:**
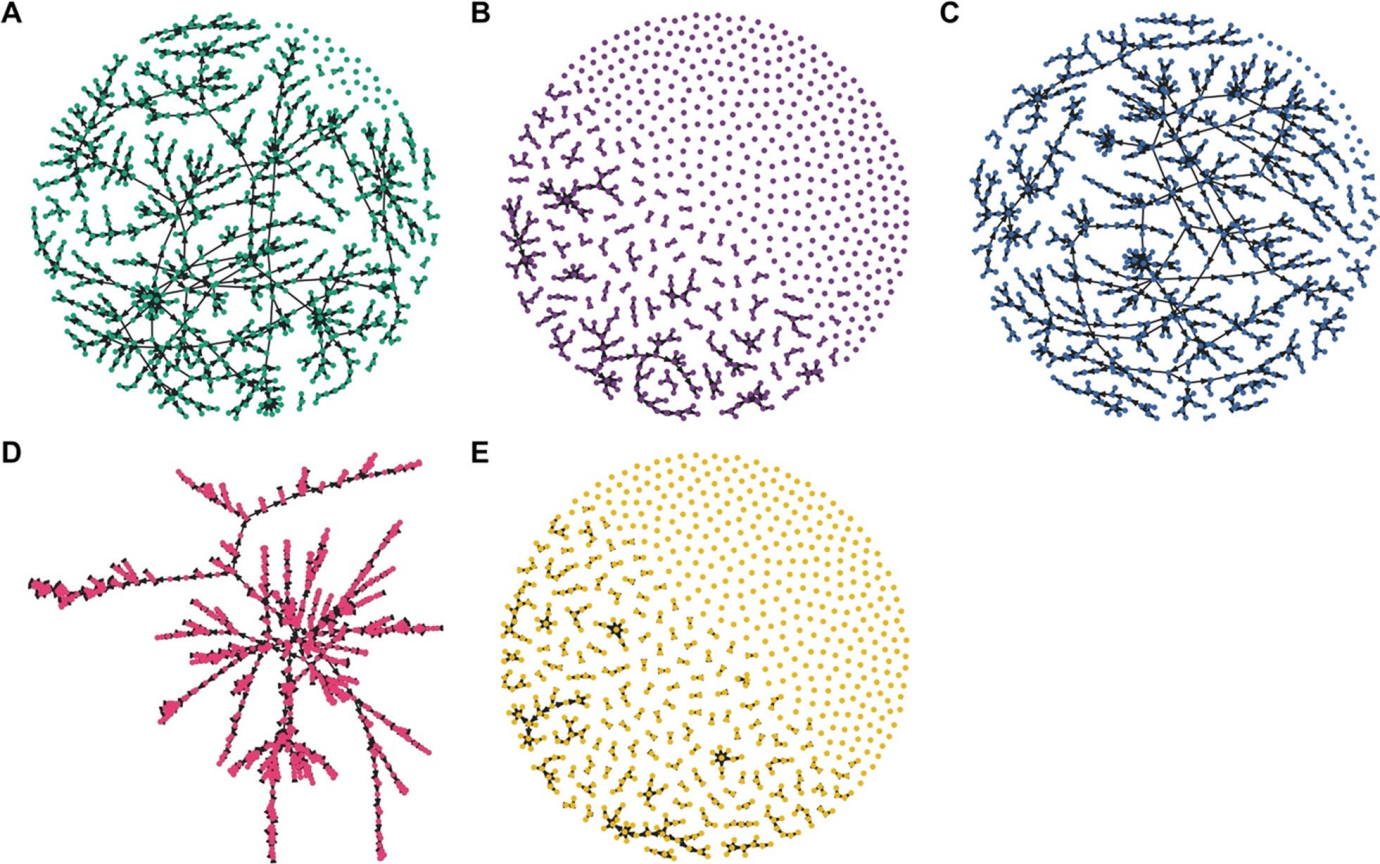
Maximum a posteriori transmission networks in Eswatini. The maximum a posteriori transmission networks (i.e., the transmission network in the posterior distribution with the highest likelihood) is shown for each inference setting: **A** spatial and temporal data while estimating the accuracy of the travel history; **B** spatial and temporal data while believing the travel history; **C** spatial and temporal data alone; **D** temporal data while estimating the accuracy of the travel history; and **E** temporal data while believing the travel history. In each transmission network, circles represent nodes, and arrows represent directed edges

Classification of cases as imported or locally acquired, key information for control programmes, was sensitive to the choice of inference setting. The proportion of cases classified as imported was most sensitive to different assumptions about the accuracy of the travel histories (Fig. 4, left column; Fig. 5). Believing the travel history yielded high estimates of importation in western Eswatini (Fig. 4C, I), whereas estimating or ignoring the travel history yielded low, relatively homogeneous estimates of importation risk (Fig. 4A, E, G). For instance, using temporal data and estimating the accuracy of the travel history produced probabilities of importation that ranged 0.0045–0.0053, suggesting that nearly all cases resulted from local transmission (Figs. 4G, 5D). Estimates of the spatial distribution of *R*_*c*_ depended most on the choice of which data types were included (Fig. 4, right column). Notably, inclusion of spatial and temporal data produced a consistent spatial distribution of relative transmission risk, with transmission hotspots in northeastern Eswatini (Fig. 4B, D, F). However, believing the travel history reduced the magnitude of transmission that was inferred from a median *R*_*c*_ of 0.95 (Figs. 4B, 5A) under default settings to 0.41 (Figs. 4D, 5B). Omitting spatial data changed the spatial distribution of transmission. Estimating the accuracy of the travel history yielded high transmission estimates (median *R*_*c*_: 1.00) in eastern Eswatini (Fig. 4H), whereas believing the travel history inferred hotspots of transmission (median *R*_*c*_: 0.42) in southern Eswatini (Fig. 4J). Believing the travel history led to slightly different median estimates of *R*_*c*_ (0.41 *vs* 0.42) depending upon whether spatial data were included, because the travel histories were unknown for 36 cases included in the analysis. As part of the inference procedure, the algorithm classified these cases as imported or locally acquired, and including spatial data caused a greater number of cases to be inferred to be imported.

### Validation of inferences from Eswatini

**Fig. 6:**
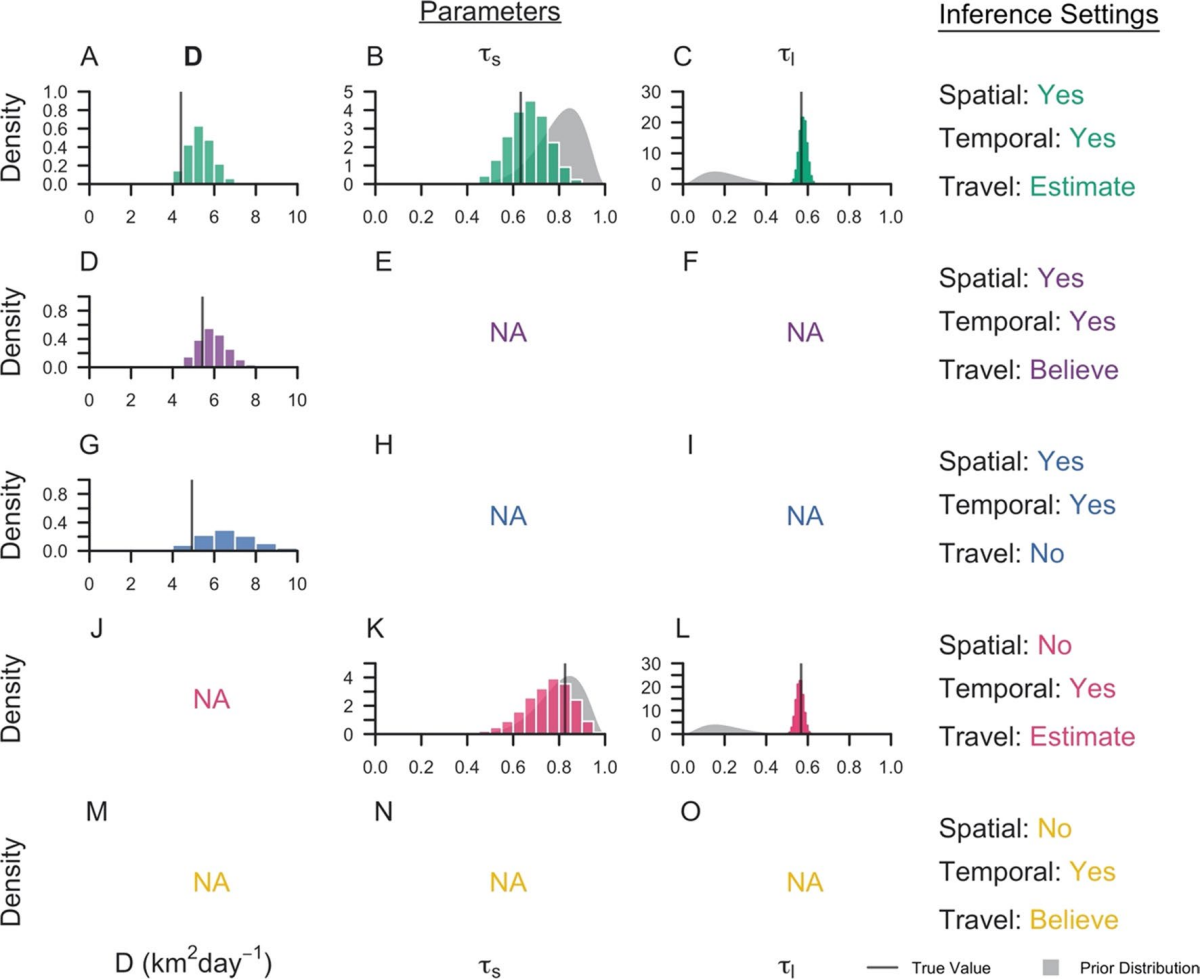
Marginal posterior distributions for parameters inferred from simulated data. The marginal posterior distributions are reported for each inference setting from its respective simulated data set. Each line denotes the true value of the parameter, and the grey shapes represent the prior distributions of the parameters. Inference settings in which a given parameter was not estimated are indicated by NA

Reconciling the different inferences under different inference settings in Figs. 2, 3, 4 and 5 was challenging because the true, underlying network and parameters were unknown. Using median posterior estimates from the Eswatini data under each inference setting, data was simulated to assess the ability of the inference method to recover the underlying parameters and transmission networks (Table 1). It was observed that the model was able to estimate the diffusion coefficient *D*, τ_s_, and τ_l_ reasonably well, depending on the inference setting (Fig. 6).

**Table 1:** Characteristics of simulated data generated using the branching process model

Inference setting			Network Size	Number of outbreaks	Prop. imported	D	τ_s_	τ_l_
Space	Time	Travel						
Yes	Yes	Estimate	775	43	0.046	4.40	0.63	0.57
Yes	Yes	Believe	775	492	0.59	5.44	1	0
Yes	Yes	No	775	36	0.039	4.93	NA	NA
No	Yes	Estimate	775	1	0.0013	NA	0.83	0.57
No	Yes	Believe	775	489	0.58	NA	1	0

A description of the simulated data used in the inference exercises are reported for each of the five inference settings. The total number of nodes in the network, the number of distinct outbreaks, the proportion of cases that are imported, and the underlying parameters are provided

**Fig. 7:**
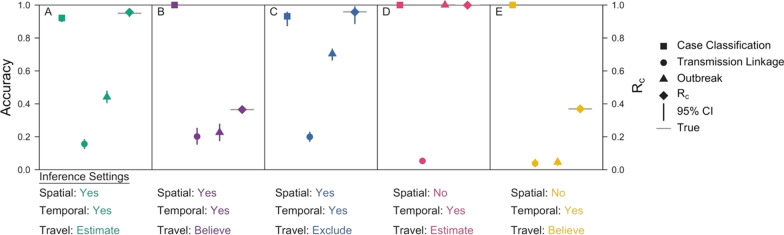
Inference accuracies for validation exercises. Accuracy metrics are reported for each inference setting applied to its respective simulated data set. Case Classification, represented by squares, refers to the proportion of cases that are correctly classified as imported or locally acquired. Transmission Linkage, denoted by circles, is the proportion of locally acquired cases for which the true parent is correctly identified. Outbreak, represented by triangles, is the proportion of locally acquired cases for which the inferred parent belongs to the correct outbreak. Bars denote the 95% credible intervals, and the grey line is the true R_c_ value of the network

The overall accuracy of classifying cases as imported or locally acquired was close to one (Fig. 7). Though seemingly promising, these high accuracies masked a tendency to overclassify cases as locally acquired, because many more cases were simulated to be locally acquired than imported. For example, under the default inference setting, the accuracy of correctly classifying imported cases was 0.15 (0.051–0.26). Similarly, the accuracies of identifying the parent of each transmission linkage were poor, despite simulating under the assumptions of the model, with accuracies ranging from 0.038 (0.017–0.063) when using temporal data and believing the travel history to 0.20 (0.16–0.25) when incorporating spatial and temporal data and believing the travel history (Fig. 7, circle points). This suggests that, as the number of cases increases within a fixed space–time window, the information content of routinely collected epidemiological data for inferring transmission chains decreases and the method becomes incapable of correctly estimating the transmission network. Nevertheless, under some settings, the method was able to capture higher-order summaries of the network, such as case classification and *R*_*c*_ (Fig. 7, square and diamond points).

### Simulation sweep

Validation of the inference algorithm revealed that its performance varied across simulated data sets. When applied to a series of simple test cases in which the transmission networks were small and in an optimal spatiotemporal arrangement, the inference method was able to reconstruct the transmission network and correctly estimate *R*_*c*_ (Additional file 1: Fig. S2). When applied to larger transmission networks in which outbreaks overlapped in space and time, performance of the inference method was poor (Fig. 7). This indicated that the performance of the inference algorithm depends on the epidemiological setting to which it is applied. To address this observation, 2,000 simulated data sets were generated in which the proportion of imported cases, the spatiotemporal window over which imported cases were distributed, the diffusion coefficient, and the accuracies of the travel history (i.e., τ_s_ and τ_l_) were varied (Additional file 1: Table S2). Then, the inference algorithm was applied under three different inference settings, and the accuracy of reconstructing each transmission network was quantified. The three inference settings used: (1) spatial and temporal data while estimating the accuracy of the travel history (default setting); (2) spatial and temporal data while believing the travel history; and, (3) spatial and temporal data alone (Additional file 1: Table S1).

**Fig. 8:**
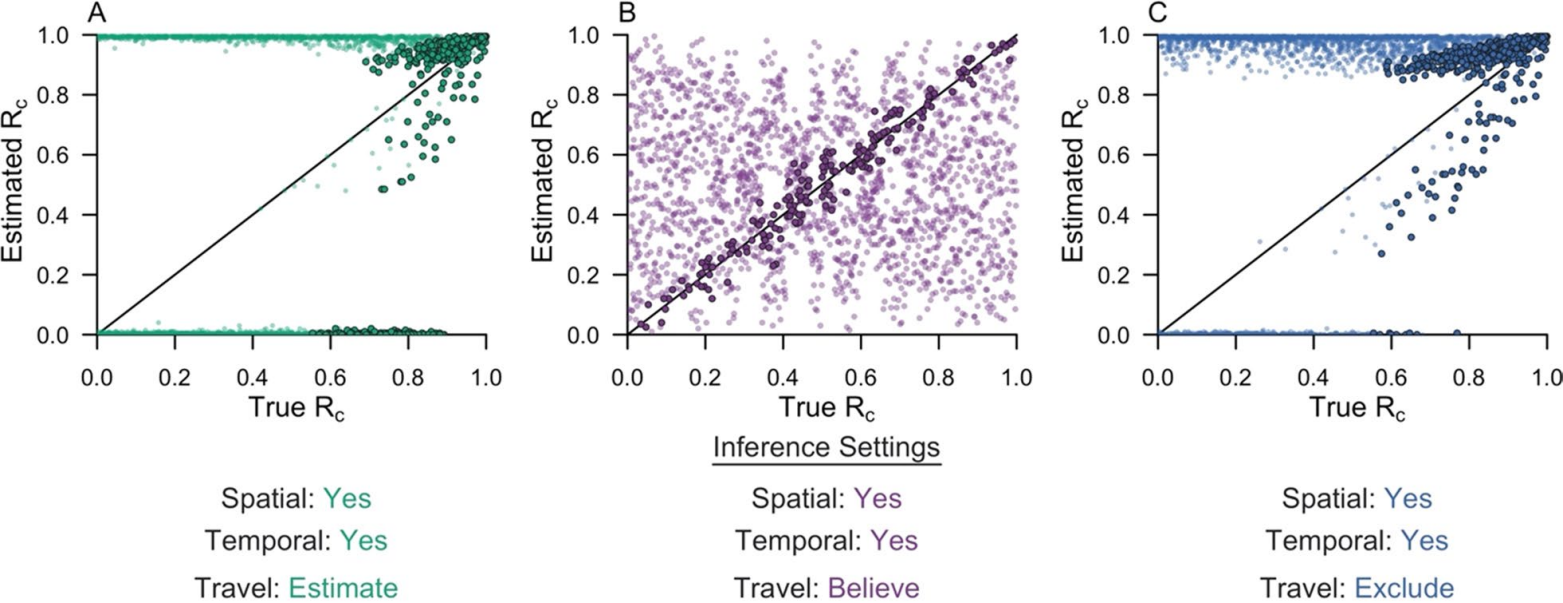
Comparison of R_c_ estimates across inference settings. The inference algorithm was applied to 2,000 simulated data sets. The estimated R_c_ is compared to the true R_c_ for each of the inference settings: **A** spatial and temporal data while estimating the accuracy of the travel history; **B** spatial and temporal data while believing the travel history; and **C** spatial and temporal data alone. Each point represents a simulated data set. The darker, accented points are simulated data sets with epidemiological features that improved performance. In **A** and **C**, the darker, accented points were simulated data sets where the mean temporal interval between imported infections was greater than two times the mean serial interval. In **B**, the darker, accented points were simulated data sets where the proportion of cases reporting travel was within 0.05 of the proportion of imported cases

The accuracy of reconstructing transmission networks depended upon both the inference setting used and the epidemiological features of the simulated data. When the algorithm used spatial and temporal data and estimated the accuracy of the travel history or excluded it, the accuracy of reconstructing transmission networks depended on the relative proportion and temporal distribution of imported cases (Additional file 1: Fig. S9 and S11). As the temporal window over which imported cases are distributed increased, the accuracy of identifying the true parent and the true outbreak of each locally acquired case increased. With an increasing temporal window, outbreaks within the transmission network became relatively more focal in time, which made the likelihoods of alternative transmission linkages more readily distinguishable. More accurate estimates of *R*_*c*_ under these inference settings similarly depended on the temporal window over which imported cases were distributed (Fig. 8A, C). When the mean temporal interval between imported infections was greater than two times the mean length of the serial interval (i.e., approximately 100 days), the estimates of *R*_*c*_ improved, although the algorithm generally overestimated it. The estimates of ${\tau }_{s}$ and ${\tau }_{l}$ also improved under these epidemiological settings (Additional file 1: Fig. S12), providing further support that the inference method can reasonably infer transmission networks under select settings. Furthermore, as the proportion of imported cases increased and *R*_*c*_ decreased, the accuracy of identifying the correct outbreak of each locally acquired case decreased (Additional file 1: Fig. S9 and S11). This pattern reflected the relationship between *R*_*c*_ and the size of individual outbreaks. As *R*_*c*_ decreased, the size of individual outbreaks decreased, and consequently, the probability that the inferred parent of a locally acquired case belonged to the same outbreak decreased (Table 2).

**Table 2:** Definitions of estimated parameters

Parameter	Definition
$D$	Diffusion coefficient (sq km day^−1^)
${\tau }_{s}$	Probability that an imported case reports travel
${\tau }_{l}$	Probability that a locally acquired case reports travel

By contrast, when the travel history was believed, the accuracy of reconstructing transmission networks depended most strongly on the accuracies of the travel history. As the probability of reporting travel increased, the accuracy of classifying imported cases increased, and the accuracy of classifying locally acquired cases decreased (Additional file 1: Fig. S10). Under this inference setting, the estimate of *R*_*c*_ depended only on the proportion of cases that reported travel. When the proportion of cases that reported travel matched the proportion of cases that were imported, *R*_*c*_ was correctly estimated (Fig. 8B).

## Discussion

The results show that, in many settings, analyses based on routinely collected surveillance data may not be capable of reconstructing individual-level transmission networks of falciparum malaria and inform estimates of the reproduction number under control, *R*_*c*_. Using simulated data similar to the Eswatini surveillance data that were analysed, the inference algorithm correctly identified transmission linkages less than 25% of the time. This inaccuracy can be primarily attributed to the inherently limited information content of spatiotemporal data on *P. falciparum* for this purpose. Its characteristically long serial interval [22] means that an appreciable number of cases presenting within a short timeframe are difficult to link to each other based on their timing, even in a relatively facile test case in which the generative process assumed in the likelihood function matched that used to simulate the data. The inability to reconstruct transmission networks using routine surveillance data has been observed for other inference algorithms when applied to pathogens, such as *Mycobacterium tuberculosis* and *Klebsiella pneuomoniae*, with similarly long serial intervals, providing further evidence that the limitations noted in this study may be generally inherent to the epidemiological data, rather than the method per se [21].

Under most simulated scenarios and assumptions about the accuracy of travel-history data, the algorithm overestimated the number of locally acquired cases, leading to overestimates of *R*_*c*_. Crucially, the simulation sweep demonstrated that routinely collected surveillance data was most informative of individual-level transmission networks and *R*_*c*_ when local outbreaks were highly focal in time. Otherwise, while the algorithm was able to reconstruct the true transmission network with modest accuracy, it tended to misclassify truly imported cases as locally acquired, thereby overestimating *R*_*c*_. Taken together, these results suggest that analyses may need to leverage additional data types beyond routinely collected surveillance data to infer transmission chains and inform fine-scale estimates of *P. falciparum* transmission in many near-elimination settings. For other purposes and at broader spatial scales, however, routinely collected surveillance data still have practical value, because the spatial distribution of cases can reveal epidemiological risk factors relevant for targeted interventions [31, 32].

Although this study was able to reach some general conclusions about the inference algorithm, the inferences were highly sensitive to which data types were included and which assumptions were made about the accuracy of travel-history data. Applying the algorithm to surveillance data from Eswatini, it was observed that inferred patterns of transmission depended on which data types were included. With the inclusion of spatial data, the inferences captured a spatial pattern of transmission consistent with another analysis from Eswatini [33] with data from a different time period. Assumptions about the travel history appeared to have a strong influence on the overall magnitude of transmission that was inferred, due to the direct relationship between *R*_*c*_ and the proportion of imported cases [16]. As a result, believing the travel history, and thereby treating it as perfectly accurate as in previous approaches [6, 18–20], could bias *R*_*c*_ estimates if there are errors in travel-history data. A study comparing community travel surveys to mobile-phone data in Kenya found that travel histories considerably underestimated the volume of travel, suggesting high rates of false negatives in community travel surveys [34]. Believing the travel history may underestimate the number of imported cases and overestimate *R*_*c*_. Accounting for inaccuracy in travel-history data is therefore important, and studies pairing community travel surveys with mobile-phone data could be used to inform prior distributions on the likely accuracy of travel-history data [34, 35].

The method that was used only considered a single spatial model to infer transmission linkages and assumed complete observation of cases, both of which are factors that could have affected our inferences based on the Eswatini surveillance data. The diffusion model that was used to represent spatial dispersion of parasites assumed that movement is isotropic in space and did not consider landscape features, such as heterogeneity in human population densities and environmental factors that may affect mosquito ecology. A study analysing self-reported movement patterns in Mali, Burkina Faso, Zambia, and Tanzania found that gravity and radiation models of spatial dispersion fit the data well, although the appropriateness of each model depended on the type of traveller, the travel distance, and the population size of the destination considered [36]. Although a variety of spatial kernels could have been used in the analysis, the conclusions reached are expected to be robust to the choice of spatial kernel, because the spatial kernel used in the likelihood matched that used to simulate the data. Regarding the representation of *P. falciparum* infections in the data set from Eswatini, there are asymptomatic and mild infections that are unlikely to have been recorded in the surveillance system yet may comprise a substantial proportion of malaria infections within Eswatini [13]. Accordingly, it is possible that the assumption of complete observation of cases could have biased *R*_*c*_ estimates, likely downward due to the fact that missing cases will tend to make offspring numbers appear smaller than they actually are [37, 38]. Even so, the conclusions about the sensitivity of transmission network inferences to the choice of data types and assumptions about travel-history data are expected to be robust to these limitations of the study. This further reinforces the conclusion of the need for caution in attempting to reconstruct person-to-person transmission networks from routine surveillance data [39], because incomplete observation of cases would lead to greater inaccuracies in our transmission network inferences beyond what was noted in the study.

Given that some of the limitations of this approach may be inherent to the information content of these data types in this system, one potential avenue for improving inferences of fine-scale patterns of *P. falciparum* transmission could involve the integration of additional data streams. For example, mobile-phone data [40], high-resolution friction surfaces [41], and other anisotropic surfaces, such as transport networks, could more realistically characterize mobility patterns and allow quantification of the effects of spatial model misspecification, whereas travel-history information that details the dates, duration and location of each trip that has been used in programmatic contexts [31] could more accurately identify importation events. Additionally, the inclusion of pathogen genetic data, which has the potential to provide a more direct signal of parasite movement, could complement traditional epidemiological data [42]. Diverse genetic markers of *P. falciparum* have been characterized in near-elimination settings, such as Eswatini [43], and have been successfully used to identify imported cases in Bangladesh [44] and Namibia [35]. There is also scope for further methodological development, such as relaxing the assumption of complete observation of infections and incorporating an underlying mechanistic model of transmission (as in Lau et al. [8]; Guzzetta et al. [45]). Incorporating an underlying mechanistic model would relax the uninformative prior assumption on all possible transmission networks, ruling out transmission networks that are epidemiologically implausible and accounting for spatial differences in transmission potential and the rate of importation due to different epidemiological and demographic factors. This approach would also permit an estimate of the serial interval distribution and seasonal variation therein directly from the data rather than borrow estimates from the literature [22, 46, 47]. To this end, this study envisions that leveraging the strengths of this method along with other, complementary methods could strengthen inferences based on routinely collected epidemiological data and open up new possibilities to make use of even more data types, such as serological data, prevalence surveys and pathogen genetic data [42, 48, 49].

## Conclusions

This study revealed limitations of analyses of routinely collected surveillance data for the inference of individual-level transmission networks of *P. falciparum*. It identified a tendency to overestimate local transmission using routinely collected surveillance data, especially when outbreaks overlapped in space and time. Using both real data from Eswatini and simulated data, this analysis identified strong sensitivities of the inferences to the epidemiological setting, the choice of data types included, and assumptions about the accuracy of travel-history data. The results indicated that using spatial and temporal data and believing travel histories yielded the most plausible estimates of transmission when applied to the Eswatini surveillance data. However, the simulation sweep demonstrated that the accuracy of the inferences strongly depended on the accuracy of the travel-history data when the travel-history data were assumed to be accurate. These sensitivities to the choice of data types and assumptions about the accuracy of travel-history data could have important programmatic implications if outputs of transmission network inferences are operationalized. Although this study was specific to *P. falciparum*, the results of the analyses indicate that future studies inferring transmission networks of *P. falciparum*, or any pathogen, should carefully consider the epidemiological setting and the choice of data types and assumptions that inform the model and should validate them using simulated data.

## Supplementary Information

**Additional file 1.** Additional figures and tables.

## Data Availability

The code and simulated data to reproduce the analyses can be found at https://github.com/johnhhuber/SpaceTime_Networks. The data collected from Eswatini contains sensitive household locations and are unable to be shared due to institutional review board restrictions.
